# Synthesis of 2-cyclopropyl-3-(5-aryl-1*H*-pyrazol-3-yl)-1,8-naphthyridine

**DOI:** 10.1186/s13588-014-0016-8

**Published:** 2014-12-21

**Authors:** Mallaiah Bucha, Laxminarayana Eppakayala, Thirumala Chary Maringanti, Giri Tharikoppula, Shashikala Kethireddy

**Affiliations:** 1 Mahatma Gandhi Institute of Technology, Gandipet, Hyderabad 50075 Telangana India; 2 Jawaharlal Nehru Technological University Hyderabad College of Engineering, Nachupally, Karimnagar, 505 501 Telangana India

**Keywords:** Grignard reagent, Cyclisation, 1,8-naphthyridine

## Abstract

**Background:**

1,8-Naphthyridine derivatives have attracted considerable attention because the 1,8-naphthyridine skeleton is present in many compounds that have been isolated from natural substances, with various biological activities.

**Findings:**

*N*,*N*-dimethoxy-*N*-methyl-1,8-naphthyridine-3-carboxamide (1) on reaction with Grignard reagent forms 2-methoxy-1,8-naphthyridine-3-carbaldehyde (2). Compound 2 on reaction with different aromatic aldehydes provided 1-(2-cyclopropyl-1,8-naphthyridin-3-yl)-3-arylprop-2-en-1-ones (3a-e) and these compounds on cyclisation with hydrazine hydrate 99% yielded 2-cyclopropyl-3-(5-aryl-1*H*-pyrazol-3-yl)-1,8-naphthyridines (4-a-e).

Synthesis of the target compounds involved the formation of 4a-e. It was accomplished using Grignard reaction, condensation reaction, and cyclisation reactions. All the synthesized compounds were readily soluble in DMSO. Spectral data of the synthesized compounds were in full agreement with the proposed structures.

**Conclusions:**

In conclusion, we have developed a simple and an efficient Synthesis of 2-cyclopropyl-3-(5-aryl-1*H*-pyrazol-3-yl)-1,8-naphthyridine.

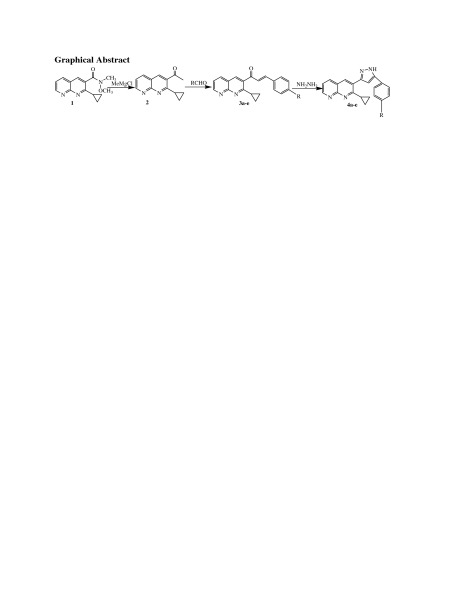

## Findings

### Background

Among the wide variety of heterocycles that have been explored for developing pharmaceutically important molecules, such as chalcones, pyrazolines and amino pyrimidines have played an important role in medicinal chemistry. The presence of reactive α,β-unsaturated carbonyl function in chalcones is found to be responsible for their antibacterial and antifungal activity. Nitrogen containing heterocyclic compounds find extensive pharmaceutical applications and possess biologically activity. Many of the naphthyridines have shown bacterial, fungicidal and carcinogenic activity [1]-[3]. As a step in this direction and in continuation of our work on 1, 8-naphthyridines [4]-[7], synthesis of the title compounds was carried out. The general synthetic procedures used in the preparation of these compounds involved the cyclisation of Schiff’s bases [8]-[12].

**Scheme 1:**
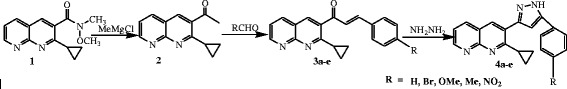
**Synthesis of 1,8-naphthyridines.**

The structure of the compounds were confirmed on the basis of their spectral (^1^H NMR and mass) data. The synthetic approach is outlined in Scheme 1.

### Methods

A simple conventional method is followed to prepare all the title compounds.

### Results and discussion

Compound (1), when reacted with Grignard reagent and gave 2-methoxy-1,8-naphthyridine-3-carbaldehyde (2) which was reacted with different aldehydes to form arylidene derivatives (3a-e), which on treatment with hydrazine hydrate afforded compound (4a-e).

### Experimental

Melting points were determined in open capillaries and are uncorrected. ^1^H NMR spectrum is taken on a Varian 500 MHz instrument with TMS as an internal standard. The chemical shifts are expressed δ ppm, and the solvent used is DMSO-d_6_. Mass spectrum is taken on Hewlett-Packard mass spectrometer operating at 70 eV. All the compounds are recrystalised from ethanol.

#### 1-(2-cyclopropyl-1,8-naphthyridin-3-yl)ethanone (2)

To a solution of 2-cyclopropyl-*N*-methoxy-*N*-methyl-1,8-naphthyridine-3-carboxamide (1) (1.01 mmol) in THF (10 mL), MeMgCl (3.03 mmol) was added drop wise at −30°C and stirred for about 2 h at −30°C. The resulting solution is quenched with a saturated NH_4_Cl solution (30 mL), filtered, extracted with EtOAc (3 × 30 mL) and dried over Na_2_SO_4_. The resulting crude compound was purified by a column chromatography by eluting 20% to 40% ethyl acetate in hexane to obtain the light yellow colour solid.

^1^H NMR (DMSO-*d*_*6*_, 500 MHz): δ 1.06-1.21 (m, 4H, 2CH2), 2.24 (s, 3H, CH_3_), 2.57 (m, 1H, CH), 7.57 (t, 1H, *J* = 12Hz CH), 8.31 (s, 1H, *J* = 12Hz CH), 8.42, (d, 1H, CH), 9.02 (d, 1H, *J* = 10Hz CH). Mass [M + 1] peak at *m*/*z* 213.

#### 1-(2-cyclopropyl-1,8-naphthyridin-3-yl)-3-phenylprop-2-en-1-one (3a)

To a solution of 1-(2-cyclopropyl-1,8-naphthyridin-3-yl)ethanone (2) (0.53 mmol) in DMF, NaOH (0.79 mmol) and aldehyde (0.63 mmol) were added at 0°C and stirred for about 1 h. Then water was added and extracted with DCM (2 × 15 mL), dried over Na_2_SO_4_ and evaporated under reduced pressure. The resulting crude compound was purified by a column chromatography by eluting 20% to 40% ethyl acetate in hexane to obtain the light yellow colour solid.

^1^H NMR (DMSO-*d*_*6*_, 500 MHz): δ 1.12-1.28 (m, 4H, 2CH_2_), 2.61 (m, 1H, CH), 7.62 (m, 2H, *J* = 14Hz 2CH), 7.81 (dd, 2H, *J* = 18Hz 2CH), 8.23, (m, 3H, 3CH), 8.51-8.61 (m, 4H, *J* = 18Hz 4CH), 9.12 (s, 1H, CH). Mass [M + 1] peak at *m*/*z* 301.

Other compounds in the series were prepared similarly and their characterization data are recorded below.

#### 3-(4-bromophenyl)-1-(2-cyclopropyl-1,8-naphthyridin-3-yl)prop-2-en-1-one (3b)

^1^H NMR (DMSO-*d*_*6*_, 500 MHz): δ 1.14-1.29 (m, 4H, 2CH_2_), 2.52,(m, 1H, CH), 7.62 (m, 4H, 4CH), 7.71 (d, 2H, *J* = 12Hz 2CH), 8.23, (d, 1H, 1CH), 8.69 (s, 1H, 1CH), 9.22 (s, 1H, CH).Mass [M + 1] *m*/*z* 380.

#### 1-(2-cyclopropyl-1,8-naphthyridin-3-yl)-3-(4-methoxyphenyl)prop-2-en-1-one (3c)

^1^H NMR (DMSO-*d*_*6*_, 500 MHz): δ 1.16-1.27 (m, 4H, 2CH2), 2.48 (m, 1H, CH), 7.62 (m, 4H, 4CH), 7.69 (d, 2H, *J* = 12Hz 2CH), 8.33 (d, 1H, , *J* = 12Hz, 1CH), 8.71 (s, 1H, 1CH), 9.24 (s, 1H, CH). Mass [M + 1] peak at *m*/*z* 331.

#### 1-(2-cyclopropyl-1,8-naphthyridin-3-yl)-3-p-tolylprop-2-en-1-one (3d)

^1^H NMR (DMSO-*d*_*6*_, 500 MHz): δ 1.19-1.28 (m, 4H, 2CH_2_), 2.53 (m, 1H, CH), 7.63 (m, 4H, 4CH), 7.72 (d, 2H, *J* = 14Hz 2CH), 8.42 (d, 1H, *J* = 14Hz, 1CH), 8.78 (s, 1H, 1CH), 9.28 (s, 1H, CH).Mass [M + 1] peak at *m*/*z* 315.

#### 1-(2-cyclopropyl-1,8-naphthyridin-3-yl)-3-(4-nitrophenyl)prop-2-en-1-one (3e)

^1^H NMR (DMSO-*d*_*6*_, 500 MHz): δ 1.23-1.31 (m, 4H, 2CH_2_), 2.62 (m, 1H, CH), 7.73 (m, 4H, 4CH), 7.81 (d, 2H, *J* = 18Hz 2CH), 8.56 (d, 1H, *J* = 16Hz, 1CH), 8.64 (s, 1H, 1CH), 9.32 (s, 1H, CH).Mass [M + 1] peak at *m*/*z* 346.

#### *2-cyclopropyl-3-(5-phenyl-1* H*-pyrazol-3-yl)-1,8-naphthyridine (4a)*

To a solution of 1-(2-cyclopropyl-1,8-naphthyridin-3-yl)-3-phenylprop-2-en-1-one (3a) (0.31 mmol) in ethanol (5 mL), hydrazine hydrate 99%(2 mL) was added. The resulting solution was refluxed for about 12 h. After completion of a starting material, the ethanol was evaporated completely under a reduced pressure then the title compound was recrystalised from diethyl ether to obtain the brown colour solid.

^1^H NMR (DMSO-*d*_*6*_, 500 MHz): δ 1.16(m, 2H, CH_2_), 1.22 (m, 2H, CH_2_), 2.21 (m, 1H, CH), 5.21(s, 1H, *J* = 12Hz CH), 5.41(s, 1H, *J* = 12Hz CH), 6.31 (m, 1H, *J* = 12Hz CH), 6.59, (m, 2H, 2CH), 7.19 (d, 1H, *J* = 10Hz CH), 7.69 (m, 4H, 4CH), 8.42 (m, 2H, 2CH), 9.11 (s, 1H, CH), 10.81 (s, 1H, NH). Mass [M + 1] peak at *m*/*z* 313.

Other compounds in the series were prepared similarly and their characterization data are recorded below.

#### *3-(5-(4-bromophenyl)-1* H*-pyrazol-3-yl)-2-cyclopropyl-1,8-naphthyridine (4b)*

^1^H NMR (DMSO-*d*_*6*_, 500 MHz): δ 1.21(m, 2H, CH_2_), 1.28 (m, 2H, CH_2_), 2.37 (m, 1H, CH), 6.21 (s, 1H, *J* = 12Hz CH), 7.32(s, 1H, CH), 7.41 (m, 4H, *J* = 12Hz 4CH), 8.19 (m, 2H, 2CH), 8.39 (d, 1H, CH), 10.69 (s, 1H, NH). Mass [M + 2] peak at *m*/*z* 392.

#### *2-cyclopropyl-3-(5-(4-methoxyphenyl)-1* H*-pyrazol-3-yl)-1,8-naphthyridine (4c)*

^1^H NMR (DMSO-*d*_*6*_, 500 MHz): δ 1.24 (m, 2H, CH_2_), 1.29 (m, 2H, CH_2_), 2.28 (m, 1H, CH), 4.12 (s, 3H, CH_3_), 6.23 (s, 1H, CH), 7.35 (s, 1H, CH), 7.29 (m, 4H, *J* = 12Hz 4CH), 8.03, (m, 2H, 2CH), 8.42 (d, 1H, CH), 10.46 (s, 1H, NH). Mass [M + 1] peak at *m*/*z* 343.

#### *2-cyclopropyl-3-(5-p-tolyl-1* H*-pyrazol-3-yl)-1,8-naphthyridine (4d)*

^1^H NMR (DMSO-*d*_*6*_, 500 MHz): δ 1.18 (m, 2H, CH_2_), 1.31 (m, 2H, CH_2_), 2.20 (m, 1H, CH), 2.39 (s, 3H, CH_3_), 6.28 (s, 1H, CH), 7.25 (s, 1H, CH), 7.39 (m, 4H, 4CH), 8.23, (m, 2H, 2CH), 8.44 (d, 1H, *J* = 12Hz, CH), 10.59 (s, 1H, NH). Mass [M + 1] peak at *m*/*z* 327.

#### *2-cyclopropyl-3-(5-(4-nitrophenyl)-1* H*-pyrazol-3-yl)-1,8-naphthyridine (4e)*

^1^H NMR (DMSO-*d*_*6*_, 500 MHz): δ 1.18 (m, 2H, CH_2_), 1.31 (m, 2H, CH_2_), 2.20 (m, 1H, CH), 6.18 (s, 1H, CH), 7.15 (s, 1H, CH), 7.27 (m, 4H, 4CH), 8.12, (m, 2H, 2CH), 8.24 (d, 1H, *J* = 12Hz, CH), 11.19 (s, 1H, NH). Mass [M + 1] peak at *m*/*z* 358.

### Conclusions

We have presented simple, cost effective, and practical method for preparation of some 1,8-naphthyridines. This methodology provides an efficient alternative to existing methods for the synthesis of 2-cyclopropyl-3-(5-aryl-1*H*-pyrazol-3-yl)-1,8-naphthyridine. Here in step-1, we observed the formation of Grignard product which on condensation of aldehydes Schiff's base is formed. Further reaction Schiff's base with hydrazine we obtained the pyrazoles.
